# ^18^F-FDG-PET-CT模拟定位在Ⅲ期非小细胞肺癌放疗中的应用研究

**DOI:** 10.3779/j.issn.1009-3419.2010.07.08

**Published:** 2010-07-20

**Authors:** 珊丹, 波 韩, 慧莹 潘, 丽娟 于, 瑞芝 王

**Affiliations:** 1 150001 哈尔滨，哈尔滨医科大学附属第一医院肿瘤放射治疗科 Department of Radiation Oncology, the First Affiliated Hospital of Harbin Medical University, Harbin 150001, China; 2 150008 哈尔滨，哈尔滨医科大学附属肿瘤医院放射治疗科 Department of Radiation Oncology, Affiliated Tumor Hospital of Harbin Medical University, Harbin 150081, China

**Keywords:** 正电子发射体层摄影, X线计算机体层摄影, 肺肿瘤, 放射治疗, PET-CT, CT imaging, Lung neoplasms, Radiotherapy

## Abstract

**背景与目的:**

PET-CT通过功能影像与解剖影像的结合，有利于更加准确地勾画非小细胞肺癌（non-small cell lung cancer, NSCLC）放射治疗靶区，PET-CT定位可能成为放疗模拟定位的新平台，而提高精确放疗的疗效。本研究试图探讨^18^F-FDG-PET-CT对CT难以界定的NSCLC放疗计划的影响。

**方法:**

对诊断明确的30例Ⅲ期NSCLC患者进行治疗体位的PET-CT扫描，扫描数据传入GE Advantage Sim 6.0放疗工作站，放疗科医生与CT和核医学科医生分别根据CT、PET-CT融合图像进行临床分期与靶区勾画，并制定相应的放疗计划。选择大体肿瘤体积（gross tumor volume, GTV）、计划靶体积（planning tumor volume, PTV）、周围正常组织受量等指标进行统计学分析。

**结果:**

① PET-CT改变临床分期：10%（3/30）的病例分期升高，10%（3/30）的病例分期降低；②PET-CT改变GTV和PTV：60%（18/30）的病例靶体积缩小，40%（12/30）的病例靶体积增大，其中体积发生明显变化者（变化 > 25%）占56.67%（17/30），但全组GTV、PTV变化不明显（*P* > 0.05）；③PET-CT改变治疗计划参数：给予相同的靶区剂量60 Gy/30次，PET参与后PTV变化者的周围正常组织受量均发生相应的变化，但各项指标全组变化不明显（*P* > 0.05）。

**结论:**

应用PET-CT模拟定位并勾画靶区，可避免靶区扩大或遗漏，对提高CT难以界定的Ⅲ期NSCLC靶区精确性有重要意义。

## Abstract (en)

**Background and objective:**

PET-CT have emerged as powerful imaging tools in radiotherapy to add information that might lead to a change in GTV definition in patients with non-small cell lung cancer (NSCLC). The aim of this study was to investigate the impact of co-registering ^18^F-FDG-PET-CT images on the gross tumor volume (GTV), the planning target volume (PTV), and critical organ dose in radiation therapy planning of NSCLC.

**Methods:**

Thirty patients with stage Ⅲ NSCLC, referred for radical radiation therapy, underwent PET-CT scanning. GTV was delineated on the CT imaging and PET-CT fused imaging respectively. The two images were used to develop the radiotherapy planning at the GE Advantage Sim 6.0 workstation. Dose-volume histograms (DVH) parameters were selected to be paralleled to compare the quality of the two plans and the impact of PET-CT on radiation plan (RP) was further analyzed.

**Results:**

(1) PET-CT image changed the clinical stage: 3 patients (10%) upstaged and 3 patients (10%) downstaged. (2) PET-CT image altered GTV and PTV: GTV and PTV was decreased by PET-CT image fusion in 18 patients (60%) and was increased in 12 patients (40%). A significant (> 25%) treatment volume modification was observed in 17 patients (56.67%). (3) PET-CT image changed the parameters of DVH: to deliver the same target dose of 60 Gy in 30 fractions, all dose-volume characteristics decreased in treatment planning for the 18 patients with decreased PTV based on PET-CT; the issue was the contrary for the 12 patients who had enlarged PTV based on PET-CT.

**Conclusion:**

The use of the co-registration of PET-CT significantly reduced inaccurate outlining of the GTV and/or to geographic misses.

精确放疗在非小细胞肺癌（non-small cell lung cancer, NSCLC）治疗中的地位已得到公认。靶区的勾画是精确放疗中最基础、最关键的一环。但CT在勾画靶区时所提供的信息有时不能满足临床要求，尤其是肺癌原发肿瘤伴有肺不张/阻塞性肺炎或确定纵隔淋巴结是否有转移时^[1]^。肺癌伴发阻塞性肺炎、肺不张，由于异常影像间有较大的重叠性，使用CT（即使是增强CT扫描）定位常难以准确划分局部肿瘤与非肿瘤的确切边界^[2, 3]^；而且CT有时不能确定短径 < 1 cm的淋巴结是否有转移。因此利用CT图像确定靶区边界有一定的局限性。对此，本研究对30例CT难以界定的局部晚期NSCLC进行PET/CT定位扫描，以探讨PET-CT模拟定位与CT定位比较对NSCLC临床分期、放疗靶区大小及周围正常组织的影响。

## 1 材料与方法

### 1.1 临床资料

选择哈尔滨医科大学附属第一医院与附属肿瘤医院2006年12月-2008年4月间收治的30例Ⅲ期NSCLC患者入组，入组标准为有细胞学或病理学明确诊断，治疗前检查临床分期为Ⅲ期，常规CT平扫表现为伴有不同程度的肺不张或可疑转移淋巴结，经增强CT扫描后依然无法准确界定肿瘤范围者。其中男性21例，女性9例，中位年龄62岁（42岁-79岁），KPS评分≥70分，无其它并发症。鳞癌12例，腺癌18例，中心型肺癌16例，边缘型肺癌14例。临床分期Ⅲa期15例，Ⅲb期15例。

### 1.2 扫描与治疗体位

患者仰卧于立体定位架内，双臂上举置于头顶，负压成型垫塑形固定体位，三维激光灯确定参考点坐标并标记于皮肤。

### 1.3 PET-CT扫描方法

应用GE公司的Discovery ST PET-CT进行检查，PET-CT、FDG合成模块均系美国GE公司出品。加速器MINI tarce生产的^18^F经Tracer Lab FN-FDG自动合成^18^F-FDG，放化纯度≥95%。所有患者均禁食6 h以上，安静休息30 min后测血糖，血糖在正常范围后按5.5 mBq/kg-7.4 mBq/kg的剂量，经肘静脉注射显像剂，避光平卧休息40 min-60 min，然后在Discovery ST PET-CT上以治疗体位平静自由呼吸状态下行CT扫描，再行PET全身断层显像。PET数据经CT衰减校正后行OSEM重建，在AW 4.2图像工作站进行PET与CT图像融合，分别得到横断、矢状和冠状的PET、CT和PET/CT图像，同时测定相应部位的CT值与PET的标准摄取值（standard uptake value, SUV）。

### 1.4 制定治疗计划

将患者的CT图像与PET图像传输至GE Advantage Sim 6.0勾画靶区以及CORVUS 6.0放射治疗计划系统，进行三维重建和图像融合，并据此进行以CT图像为基础和以PET/CT融合图像为基础的放疗计划设计。

#### 1.4.1 大体肿瘤体积GTV_CT_（gross tumor volume, GTV）定义

放疗科医生参考CT诊断医生的意见，参照2002 AJCC肺癌分期标准进行临床分期，并结合胸部增强CT扫描影像，在GE Advantage Sim 6.0治疗计划系统CT扫描横断面上逐层勾画，定义为GTV_CT_，原发灶靶区在肺窗条件（窗位800 Hu，窗宽1 600 Hu）勾画；转移淋巴结靶区在纵隔窗条件（窗位40 Hu，窗宽400 Hu）勾画，将最短径 > 1 cm，在增强扫描CT上不增强或轻度增强定义为转移淋巴结并进行勾画。

#### 1.4.2 GTV_PET/CT_定义

在GE Advantage Sim 6.0工作站进行PET与CT图像的融合，放疗科医生与核医学和CT医生联合阅读PET、CT及PET-CT融合图像，依据融合图像重新进行临床分期，通过观察CT所示病变部位大小、形态、与周围组织关系、CT值和相应位置PET所示FDG代谢情况，在放射性浓聚灶显示最清楚的层面上勾画感兴趣区（region of interest, ROI）。原发肿瘤PET显像应用目测法与SUV值半定量方法（SUV > 2.5）综合判定定义PET显像阳性，最后通过融合图像结合增强CT扫描结果进行准确定位，勾画GTV_PET-CT_。如果PET扫描病变边界大于CT，以PET上病变确定GTV_PET-CT_；如果PET扫描病变边界小于CT，则参考增强CT扫描图像与SUV值定义GTV_PET-CT_；转移淋巴结以目测法分析图像，当淋巴结浓聚程度高于纵隔血池为阳性判断标准，对CT图像上最短径≤1 cm而PET阳性的淋巴结，视为转移淋巴结。

#### 1.4.3 临床靶体积（clinical tumor volume, CTV）和计划靶体积（planning tumor volume, PTV）的定义

靶区定义按照国际辐射单位与测量委员会（International Commission on Radiation Units, ICRU）50号和62号文件规定标准定义，CTV范围包括原发灶（边界为肺窗条件下GTV鳞癌、腺癌分别外放0.5 cm、0.7 cm）及纵隔转移淋巴结（外放0.5 cm），不做纵隔淋巴引流区预防照射。原发肿瘤的PTV在头足（SI）、左右（RL）方向的移动度依据治疗体位模拟透视下观察的肿瘤移动幅度而定，在前后（AP）方向外放范围参考文献而设定外放5 mm；转移淋巴结则是在CTV基础上各方向外放5 mm。

#### 1.4.4 处方剂量的设计及治疗计划的优化

应用CORVUS 6.0治疗计划系统进行计划设计与剂量计算。方案优化标准为：靶区剂量60 Gy/30次。应用组织不均匀性校正，90%等剂量线包绕PTV，肺V20（percentage of lung receiving RT dose more than 20 Gy, V20） < 32%，双肺平均剂量（mean lung dose, MLD）≤18 Gy，食管V55（percentage of esophagus receiving RT dose more than 55 Gy, V55）≤28%，V45（percentage of esophagus receiving RT dose more than 45 Gy）≤40%，脊髓最大受照剂量Ds≤45 Gy，全心平均剂量（mean heart dose, MHD）≤30 Gy。危险器官如食管、脊髓、双肺和心脏也被勾画。

### 1.5 观察指标

① GTV_CT_和GTV_PET-CT_的体积，PTV_CT_和PTV_PET-CT_的体积；②基于不同PTV的放疗计划的正常器官体积剂量直方图DVH（dose volume histogram）分布的差异。

### 1.6 统计方法

应用SPSS 13.0软件，靶区大小经检验后符合正态分布采用配对*t*检验，参数大小经检验为非正态分布采用秩和检验。

## 2 结果

### 2.1 分期改变

30例中6例PET-CT检查后分期改变。3例分期下降，其中2例为PET排除了CT诊断为转移的纵隔淋巴结，TNM分期从T3N2M0改为T3N0M0，临床分期从Ⅲa期改为Ⅱb期，1例是PET排除了对侧纵隔转移淋巴结，TNM分期从T3N3M0期改为T3N2M0期，临床分期从Ⅲb期降为Ⅲa期。3例分期升高者，2例因PET发现了对侧肺门和对侧纵隔转移淋巴结使TNM分期从T2N2M0改为T2N3M0，临床分期从Ⅲa期改为Ⅲb期，1例PET/CT证实肿瘤累及心包，使TNM分期从T2N2M0改为T4N2M0，临床分期从Ⅲa期改为Ⅲb期。

### 2.2 ^18^F-FDG-PET-CT显像对NSCLC的靶区体积的影响

所有病例经PET-CT扫描后靶区体积均发生了变化，GTV_PET-CT_ < GTV_CT_者18例（GTV_PET-CT_=100.97±44.22, GTV_CT_=162.53±66.29, *t*=4.101, *P*=0.001），该18例中仅3例是由于排除了转移淋巴结使靶区缩小，其余15例均为PET-CT明确了肺不张与肿瘤的边界，排除了误认为是肿瘤的不张的肺组织，其中靶区变化明显者（变化大于25%）11例（61.1%）；GTV_PET-CT_ > GTV_CT_者12例（GTV_CT_=118.45±70.07, GTV_PET-CT_=170.58±72.58, *t*=2.887, *P*=0.015），均由于PET-CT检查定位后发现了更多的转移淋巴结，其中淋巴结靶区变化明显者（变化 > 25%）6例（50.0%），但全组GTV变化不明显（GTV_PET-CT_=128.81± 65.93, GTV_CT_=144.89±70.15, *t*=1.047, *P*=0.304）（图 1）。

**1:**
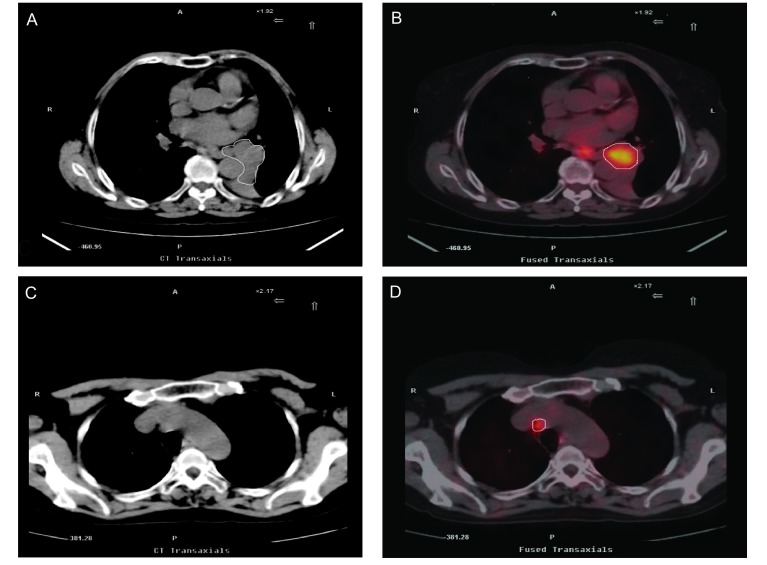
^18^F-FDG-PET/CT显像对NSCLC的靶区体积的影响。A：CT示左肺下叶癌并肺不张；B：融合图像区分了不张与肿瘤组织；C：CT示无明确的转移淋巴结；D：PET-CT发现相应部位淋巴结放射性浓聚。 The Influence of PET-CT image on tumor volume of NSCLC. A: CT showed primary tumor complicated with atelectasis of left lower lobe of the lung; B: PET-CT showed activity uptake of gross tumor; C: CT showed negative mediastinal lymph node; D: PET-CT showed activity uptake of mediastinal lymph node.

所有病例的PTV均发生相应的改变，18例缩小（PTV_PET-CT_=192.85±89.68, PTV_CT_=285.73±96.97, *t*=3.803, *P*=0.003），12例增大（PTV_PET-CT_=281.43±110.38, PTV_CT_ =188.04±72.50, *t*=3.806, *P*=0.001），但全组PTV变化不明显（PTV_PET-CT_=227.12±94.96, PTV_CT_=245.99±110.19, *t*=0.780, *P*=0.442）。

### 2.3 对周围正常组织的影响

全组病例CT计划与PET-CT计划的DVH参数比较，所有参数两个计划均无明显差异（*P*值均 > 0.05），但将全组病例分为PTV_PET-CT_ < PTV_CT_与PTV_PET-CT_ > PTV_CT_两个亚组后，放疗计划DVH参数的比较则有统计学差异。

#### 2.3.1 PTV_PET-CT_ > PTV_CT_组

共有12例PTV_PET-CT_ > PTV_CT_的患者，基于PET-CT融合图像的放疗计划与基于CT图像的放疗计划相比，在给予相同肿瘤靶区剂量60 Gy时，肺食管脊髓和心脏等正常器官的受量显著增加（*P*值均 < 0.05，表 1）。

**1:** PET-CT定位PTV增大者正常组织DVH参数比较 DVH parameters of normal tissue for increased PTV based on PET-CT

DVH index	CT plan	PET-CT plan	*Z*	*P*
Lung V10 (%)	47.12±10.81	54.06±9.15	2.275	0.023
Lung V20 (%)	20.87±5.89	26.36±4.88	3.059	0.002
Lung V40 (%)	8.16±2.29	10.45±0.99	3.059	0.002
Lung MLD (cGy)	13.22±2.60	14.54±1.95	2.197	0.028
Esop MED (cGy)	13.15±2.76	14.94±2.68	3.059	0.002
Spinal Ds (cGy)	20.35±6.54	23.92±9.37	3.059	0.002
Hear MHD (cGy)	12.83±4.39	16.90±2.98	3.059	0.002

#### 2.3.2 PTV_PET-CT_ < PTV_CT_组

共有18例PTV_PET-CT_ < PTV_CT_的患者，基于PET-CT融合图像的放疗计划与基于CT图像的放疗计划相比，在给予相同肿瘤靶区剂量60 Gy时，肺食管脊髓和心脏等正常器官的受量显著降低（*P*值均 < 0.05，表 2）。

**2:** PET-CT定位PTV缩小者正常组织DVH参数比较 DVH parameters of normal tissue for decreased PTV based on PET-CT

DVH index	CT plan	PET-CT plan	*Z*	*P*
Lung V10 (%)	58.52±12.20	51.24±17.16	3.724	< 0.001
Lung V20 (%)	37.86±7.38	28.05±6.54	3.724	< 0.001
Lung V40 (%)	12.10±2.51	8.88±2.12	3.724	< 0.001
Lung MLD (cGy)	18.39±1.74	15.45±2.77	3.680	< 0.001
Esop MED (cGy)	19.91±3.72	16.69±3.39	3.506	< 0.001
Spinal Ds (cGy)	31.47±6.99	25.78±5.62	3.724	< 0.001
Hear MHD (cGy)	15.04±4.66	11.49±4.54	3.724	< 0.001

## 3 讨论

PET-CT在恶性肿瘤诊治过程中的应用越来越广泛，其所发挥的作用也日益引人嘱目。PET-CT检查相对于目前常用的CT、MRI等常规影像更能清楚直观地确定肿瘤部位、淋巴结状态、远处转移的范围，在精确肿瘤的临床分期、确定病变范围等方面日益显示出优势，为选择最佳的综合治疗方案提供了有力保障。

PET-CT用于放疗模拟定位，能提供更丰富和准确的图像信息^[4-6]^。目前临床上定义NSCLC放疗靶区通常是以CT影像为基础的，但必须认识到以反映解剖结构和组织密度等形态学改变为主的CT扫描对于合并肺不张的NSCLC在确定肺不张与局部病变的相互关系上有一定的局限性。对于此类患者进行精确放疗靶区勾画时具有一定的盲目性，即便是增强CT扫描作用亦有限，为防止遗漏肿瘤组织，往往把过多的正常肺组织划入GTV，导致GTV体积夸大，不仅增加了放疗并发症发生率，同时也影响局部肿瘤控制率和患者生存率^[7]^。

PET-CT扫描用于NSCLC放射治疗模拟定位，改变了NSCLC患者的临床分期。国内巩合义等^[8]^对58例NSCLC进行PET-CT检查，其中36.2%患者的临床分期发生改变，27.6%的患者治疗计划发生改变。本研究对30例NSCLC患者进行PET-CT检查，20.0%（6/30）的病例临床分期发生改变，比例低于其它相关研究，原因可能是本组研究选择的均是Ⅲ期病例，分期集中，PET发现更多的转移淋巴结多为期内改变，治疗决策未发生改变，但所有病例的放射治疗计划均发生了改变。

国内外许多相关研究结果证实PET显像对合并肺不张的NSCLC具有重要的临床价值。Pieterman^[9]^报道PET显像对于肿瘤组织和膨胀不全的肺组织，其SUV值有明显差异，研究者认为膨胀不全的肺组织代谢活性很低，即根据SUV值的不同，可以区分肿瘤组织和周围性肺不张。Bakheet^[10]^报道PET显像具有一定的假阳性，限制了PET的特异性，最主要的原因就是炎症或感染因素。因而建议在此类患者中宜先行抗炎治疗，以尽量避免假阳性的干扰。

PET显像结合CT等影像学资料对于NSCLC原发肿瘤放疗范围的确定具有重要的临床意义和价值。Bradley等^[11]^对26例NSCLC患者分别行PET-CT和CT检查，并为每个病例分别勾画两个GTV，结果显示：PET-CT融合图像因为避开了CT上认为是肿瘤的阻塞性肺炎或肺不张组织，而使3例患者的GTV降低。本组病例经PET-CT扫描后GTV缩小者18例，其中多数83.3%（5/18）是由于PET扫描区分了肿瘤组织与肺不张，有效地降低周围正常组织的放射损伤，提高了治疗精度。

在淋巴结靶区勾画方面，CT常以淋巴结大小作为诊断是否转移的标准，但是不同部位正常淋巴结大小不一，炎性或反应性增生可造成淋巴结增大，而且约15%的转移淋巴结也无明显体积变化^[12]^，因此，CT定义淋巴结靶区存在一定的局限性^[1]^。^18^F-FDG-PET-CT显像在检出纵隔转移淋巴结方面优于CT，其灵敏性、特异性、准确性分别为96%、93%和94%，而CT为68%、65%和66%，尤其在检出 < 1.0 cm阳性淋巴结方面显示出优势，灵敏性、特异性及准确度为80%、95%、92%^[13]^。本组研究中12例患者因PET-CT发现了更多的转移淋巴结使GTV增大，其中50.0%（6/12）的GTV-N变化明显（变化 > 25%）；靶区缩小的18例患者中3例是由于排除了CT诊断的转移淋巴结；16.6%（2/12）的患者通过PET扫描发现更多的转移淋巴结使N分期升高。

PET/CT检查后靶区体积改变也相应的影响了周围正常组织的受照剂量和受照体积。Elisabeth等^[14]^比较行适形放疗的NSCLC患者的CT计划和PET-CT计划，51.7%的患者GTV发生 > 25%的明显改变，其放疗计划的V20、V36（全心脏受量 > 36 Gy）也发生了明显改变。张碧媛等^[15]^报道15例NSCLC接受PET-CT检查后，7例因检出更多的转移淋巴结而使靶区增大（*P* < 0.001），另外8例排除了部分CT阳性淋巴结而使靶区缩小（*P* < 0.001），靶区增大及缩小者的V20、MLD、食管V45、V55、MHD等均显著提高或降低。巩和义等^[8]^的研究同样发现在伴有肺不张和阻塞性肺炎时可明显缩小GTV，可更好地保护周围正常肺组织。

本研究的结果与相关研究相似，比较两个放疗计划，根据PET-CT融合图像勾画靶区使靶区体积均发生了变化。40.0%（12/30）因检出更多的转移淋巴结而使靶区增大，避免了靶区遗漏带来的局控率的降低；60.0%（18/30）靶区体积缩小，其中多数83.3%（15/18）是由于PET扫描区分了肿瘤组织与肺不张，有效地降低周围正常组织的放射损伤，提高了治疗精度；所有病例中靶区体积发生明显变化者占56.6%（17/30）。靶区的改变使V20、V40、MLD、MED、MHD、Ds等指标发生了相应的变化。

综上所述，^18^F-FDG-PET-CT指导非小细胞肺癌的放射治疗，可以更加准确定义靶区，特别是对CT图像上难以界定性质或范围的病变，PET-CT提供了更准确的判断依据，有效避免了靶区遗漏或盲目扩大，为非小细胞肺癌精确放疗提供了有效的新方法。应该注意的是，利用PET-CT融合图像定义NSCLC靶区也并非尽善尽美，如NSCLC原发肿瘤病灶在单纯PET显像上定义病灶边界尚无理想的方法，应用PET/CT融合图像确定原发肿瘤GTV边界尚需进一步研究。同时，^18^F-FDG-PET-CT显像在定性诊断NSCLC的淋巴结方面也存在一定的假阴性与假阳性，因此^18^F-FDG-PET-CT检查作为一种新的影像技术，应用于NSCLC的放射治疗过程，需要更细致、更深入的研究。
